# The TLR4/TRIF-Mediated Activation of NLRP3 Inflammasome Underlies Endotoxin-Induced Liver Injury in Mice

**DOI:** 10.1155/2010/641865

**Published:** 2010-06-16

**Authors:** Hiroko Tsutsui, Michiko Imamura, Jiro Fujimoto, Kenji Nakanishi

**Affiliations:** ^1^Department of Microbiology, Hyogo College of Medicine, 1-1, Mukogawa-cho, Nishinomiya 663-8501, Japan; ^2^Department of Surgery, Hyogo College of Medicine, 1-1, Mukogawa-cho, Nishinomiya 663-8501, Japan; ^3^Department of Immunology & Medical Zooloty, Hyogo College of Medicine, 1-1, Mukogawa-cho, Nishinomiya 663-8501, Japan; ^4^Cancer Center, Hyogo College of Medicine, Nishinomiya, Japan

## Abstract

Administration of heat-killed *Propionibacterium acnes* renders mice highly susceptible to LPS. After LPS challenge *P. acnes*-primed mice promptly show hypothermia, hypercoagulation (disseminated intravascular coagulation), elevation of serum proinflammatory cytokine levels, and high mortality. The surviving mice develop liver injury. As previously reported, IL-18 plays a pivotal role in the development of this liver injury. Many cell types including macrophages constitutively store IL-18 as biologically inactive precursor (pro) form. Upon appropriate stimulation exemplified by TLR4 engagement, the cells secrete biologically active IL-18 by cleaving pro-IL-18 with caspase-1. Caspase-1 is also constitutively produced as a zymogen in macrophages. Recently, NLRP3, a cytoplasmic pathogen sensor, has been demonstrated to be involved in the activation of caspase-1. Here, we review the molecular mechanisms for the liver injuries, particularly focusing on the TLR4/NLRP3-mediated caspase-1 activation process, with a brief introduction of the mechanism underlying *P. acnes*-induced sensitization to LPS.

## 1. Introduction

TLR, topics of this issue, is an extracellular sensor family of pathogen-associated molecular patterns (PAMPs) [1, 2]. As described by Yamamoto et al. in this special issue, TLR1, TLR2, TLR4, TLR5, TLR6, and TLR11 are expressed on the cell surface, while TLR3, TLR7, TLR8, and TLR9 are expressed on the membrane of endosome, which is a transport vesicle originated from the cell membrane to trap and transport the extracellular macromolecules into the inside of the cells. Besides, mammalian host possess cytoplasmic sensors consisting of at least two families, RIG-I-like receptor (RLR) and Nod-like receptor (NLR) families [3–5]. After sensing intracellular, virus-derived double-stranded (ds) RNA, RLR members relay a signal to activate inflammatory responses for viral clearance via induction of proinflammatory cytokines and type 1 IFNs [6, 7]. Some of the NLR family members are associated with the cytoplasmic formation and activation of inflammasome. Inflammasome is a multiple protein complex and is regarded as the platform for activation of caspase-1 [8, 9]. Caspase-1 is produced as enzymatically inactive precursors (pro) and requires appropriate cleavage to become active. Macrophages including Kupffer cells constitutively produce procaspase-1 and accomplish caspase-1 activation in the inflammasomes after being stimulated [10–12]. Caspase-1 cleaves biologically inactive pro-IL-1*β* and pro-IL-18, leading to extracellular release of the corresponding active forms. Many cell types including Kupffer cells produce and store pro-IL-18 in the steady state, while they start to produce pro-IL-1*β* only after activation with appropriate stimuli [13–15]. Thus, the inflammasomes contribute to the secretion of IL-18 and IL-1*β* via activation of caspase-1.

**Figure 1:**
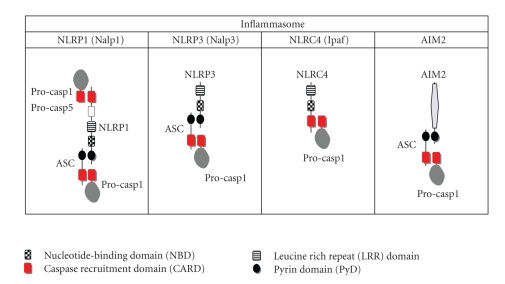
Inflammasome constituents. There have been reported at least four types of inflammasomes, NLRP1 (Nalp1), NLRP3 (Nalp3), NLRC4 (Ipaf), and AIM2 inflammasomes. NLR is subdivided into two groups, PYD-possessing group, namely, NLRP and PYD-lacking group NLRC. After exposure of cells to the corresponding stimuli, these NLRs and AIM2 are believed to be self-oligomerized. As it contains CARD at an N-terminus and PYD at a C-terminus, self-oligomerized NLRP1 can recruit procaspase (casp)-1 and procaspase-5 by action of its CARD and also assembly procaspase-1 with help from ASC that possesses both PYD and CARD. Self-oligomerized NLRP3 recruits procaspase-1 by interposing ASC between them. In contrast, self-oligomerized NLRC4 recruits procaspase-1 by directly interacting CARD of procaspase-1 with its CARD. AIM2, belonging a different protein family, is also believed to be self-oligomerized after recognition of double-stranded DNA and recruits procaspase-1 with help from ASC. NLR, Nod-like receptor; CARD, caspase recruitment domain; PYD, pyrin domain.

**Figure 2:**
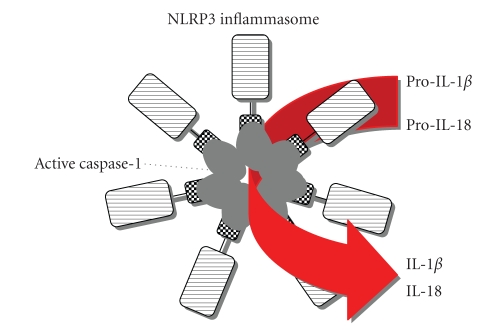
A possible schema for IL-18/IL-1*β* processing by the inflammasome. After appropriate stimulation, the NLRP3 inflammasome is activated to induce active caspase-1, which eventually results in conversion of pro-IL-18/pro-IL-1*β* into biologically active IL-18/IL-1*β*.

Inflammasome is composed of certain member of NLR and procaspase 1 [5, 8, 9] (Figure 1). NLR family members are divided into two groups. One is an NLRP group possessing pyrin domain (PYD), and the other is an NLRC group lacking PYD but possessing caspase recruitment domain (CARD) [19]. NLRP1 (Nalp1), NLRP3 (Nalp3), and NLRC4 (Ipaf) have been demonstrated to nucleate the inflammasomes [5, 19]. In the inflammasome these NLRs are believed to sense cytoplasmic PAMPs exemplified by LPS presumably via their leucine rich repeat (LRR) domain. LRR domain of NLRP3 can recognize all of the TLR agonists except for TLR5 agonist, flagellin of bacterial flagellum, while that of NLRC4 senses flagellin [20]. After being stimulated the NLRs are self-oligomerized by binding each other using their nucleotide-binding domain (NBD). Self-oligomerized NLRP1 directly recruits procaspase-1 by homophilic protein-protein interaction between its N-terminal CARD and CARD of procaspase1 and/or procaspase-5 [8]. ASC consisting of PYD and CARD is regarded as an adapter protein for caspase-1 activation. The NLRP1 can bind to PYD of ASC by its PYD domain at C-terminus, and CARD of ASC eventually recruits procaspase-1 by CARD-CARD interaction (Figure 1). The same scenario can be sketched for the recruitment of procaspase-1 around the oligomerized NLRP3-ASC complexes (Figure 1). NLRC4 has CARD but not PYD. Upon appropriate stimulation of NLRC4, procaspase-1 is recruited onto NLRC4 directly by CARD-CARD interaction (Figure 1). Recently, AIM2, belonging to a different protein family namely PYHIN, was reported to activate caspase-1 by sensing cytoplasmic ds-DNA [7, 21–25]. After recognition of ds-DNA by HIN 200 domain of it, AIM2 might be self-oligomerized for recruitment of procaspase-1 by similarly interposing ASC between these two proteins (Figure 1). Recruitment of procaspase-1 into these inflammasomes is likely to activate caspase-1, leading to conversion from pro-IL-18 and pro-IL-1*β* into active IL-18 and IL-1*β* [26] (Figure 2). 

As previously reported, mice having received heat-killed *Propionibacterium acnes* are highly susceptible to LPS [27–30]. *P. acnes*-primed mice suffer from liver injuries after LPS challenge. However, administration of neutralizing anti-IL-18 antibodies (Abs) just before LPS challenge can prevent *P. acnes*-primed mice from the liver injury [17]. Besides, *Il18^−/−^* mice are resistant to the *P. acnes*/LPS treatment [18]. Thus, IL-18 is important for the development of liver injuries. Here, we review the mechanisms for the *P. acnes*/LPS-induced liver injuries, particularly focusing on those how active IL-18 is released. Prior to addressing this, we would like to introduce the cellular and molecular mechanisms by which pretreatment with *P. acnes* render mice highly susceptible to LPS.

## 2. Endotoxin Shock Syndrome in *P. acnes*/LPS-Treated Mice

Hypothermia, hypercoagulation (disseminated intravascular coagulation; DIC), high lethality, and tissue injuries are major clinical manifestations of endotoxin shock syndrome [30–35]. After challenge with a subclinical dose of LPS, naïve wild-type (WT) mice do not show these signs (Table 1). In contrast, mice having received heat-killed *P. acnes* 7 days before are highly susceptible to LPS. *P. acnes*-primed mice, but not naïve mice, show obvious and gradual reduction of rectal temperature, serum elevation of proinflammatory cytokines including IL-6, IFN-*γ*, and TNF-*α*, high mortality and liver injuries after challenge with the same subclinical dose of LPS [18, 30, 32]. LD_50_ to LPS in *P. acnes*-primed mice is a thousandth or less of that in naïve mice [30]. Furthermore, they exhibit severe hypercoagulation status, which is monitored by plasma levels of coagulation indicator, thrombin antithrombin complexes (TAT), and anti-fibrinolytic protein, plasminogen activator type 1 (PAI-1) that potently inhibits fibrinolysis by blocking conversion from plasminogen into fibrinolytic plasmin [32, 36]. *P. acnes*-primed mice, but not naïve mice, tremendously increase plasma levels of TAT and PAI-1 after LPS challenge [32]. Thus, *P. acnes* treatment powerfully sensitizes mice to LPS.

## 3. Kupffer Cell Ingestion of Heat-killed *P. acnes*

**Figure 3:**
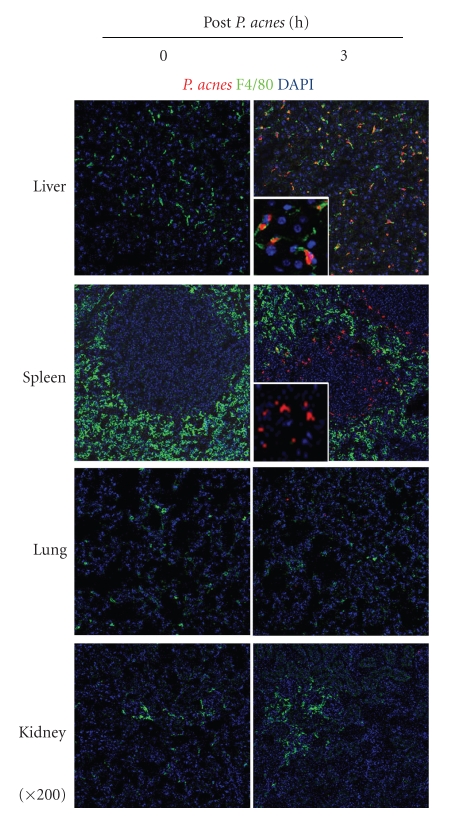
Kupffer cells promptly capture heat-killed *P. acnes*. Cy3-labeled, heat-killed *P. acnes* (red) were administered into naïve wild-type mice through a tail vein, and at 3 h tissue specimens were sampled. Frozen tissue slices were incubated with anti-F4/80 mAb (green) and DAPI for detecting macrophages and cell nuclei, respectively. F4/80^+^ cells (Kupffer cells) capture *P. acnes*, while both F4/80^+^ and F4/80^−^ cells ingest them in the spleen. In contrast to live and spleen, both lung and kidney rarely contain *P. acnes*. Magnification of insets is x800.

*P. acnes*, a Gram-positive bacterium, is often detectable on human skin and has been believed to be relevant to various inflammatory diseases, such as synovitis, acne, pustulosis, hyperostosis, and osteitis (SAPHO) and sarcoidosis [37]. What happens in mice treated with heat-killed *P. acnes*? To address this, we labeled heat-killed *P. acnes* by Cy3, injected them into WT mice through a tail vein, and sampled various tissue specimens at 3 h. We examined tissue distribution of Cy3^+^ particles by confocal microscopic analyses. Expectedly, heat-killed *P. acnes* are accumulated in the liver and spleen, whereas they were almost absent in the lung and kidney (Figure 3). F4/80^+^ cells principally capture *P. acnes* in the liver, while both F4/80^−^ cells and F4/80^+^ cells ingest them in the spleen (Figure 3). 

**Figure 4:**
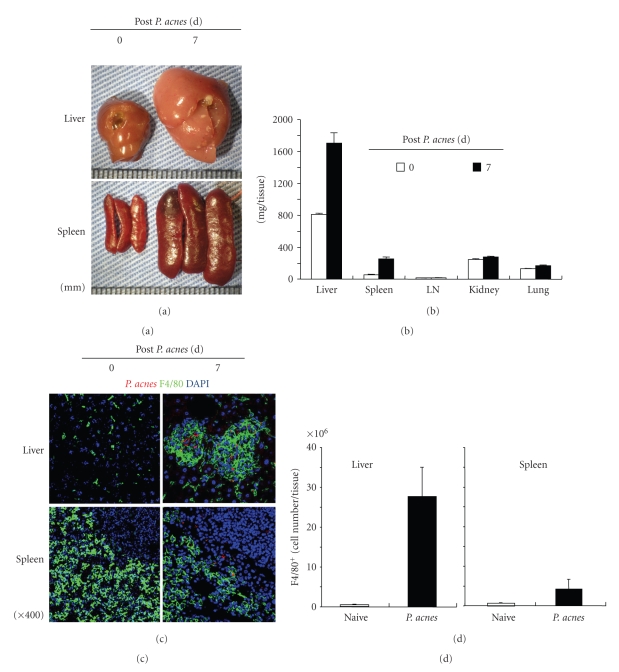
Hepatosplenomegaly and hepatic granulomas in *P. acnes*-primed mice. Wild-type mice received Cy3-labeled, heat-killed *P. acnes*, and at day 7 various tissues were removed and weighed (a, b). The liver slices were stained with anti-F4/80 mAb and DAPI for detecting macrophages and cell nuclei, respectively (c). Kupffer cells and splenocytes were prepared from *P. acnes*-primed (closed) or naïve mice (open). After staining with anti-F4/80 mAb, proportion of F4/80^+^ cells were determined by FACS, and total F4/80^+^ cell number was counted.

At day 7 after *P. acnes* treatment tremendous hepatosplenomegaly is observed (Figure 4(a)). The liver doubles its normal weight, whereas weight of spleen achieve more than 5 times (Figure 4(b)). In contrast to the liver and spleen, weight of kidney or lung remains unchanged. In the liver, the dense granulomas primarily consisting of F4/80^+^ macrophages develop, in the center of which *P. acnes*-ingested F4/80^+^ Kupffer cells are localized (Figure 4(c)), suggesting that *P. acnes*-ingested F4/80^+^ Kupffer cells might recruit many F4/80^+^ macrophages. Immunostaining using rhodamine-conjugated anti-F4/80 mAb followed by counter-staining with hematoxylin reveals that abundant F4/80^+^ cells are present in the hepatic granulomas [38]. In contrast to the liver, obvious accumulation of F4/80^+^ cells around *P. acnes* is absent in the spleen (Figure 4(c)). Many dendritic cells were reported to compose the hepatic granulomas as well [39, 40]. *P. acnes* treatment increases hepatic F4/80^+^ cell number to 30 times and more of that in naïve mice, while the splenic F4/80^+^ cell number reaches only less than 5 times (Figure 4(d)). Furthermore, splenic macrophages from *P. acnes*-primed mice produce much higher levels of proinflammatory cytokines including TNF-*α* in response to LPS than do those from naïve mice [32]. This is also the case for Kupffer cells. Thus, *P. acnes* treatment induces both numerical increase and qualitative alteration of macrophages in the liver and spleen. This may implicate the importance of macrophages for the accomplishment of the LPS sensitization by *P. acnes* treatment.

## 4. Requirement of Macrophages for the Sensitization to LPS Induced by *P. acnes* Treatment

Depletion of macrophages rescues *P. acnes*-primed mice from the liver injury and high mortality induced by the subsequent challenge with LPS [38] (Table 1). This clearly demonstrates the indispensability of macrophages for the *P. acnes*-induced sensitization to LPS. Intravenous injection of clodronate liposome depletes macrophages in mice, while control PBS liposome do not affect them [41]. These two groups of mice are treated with *P. acnes*, followed by LPS challenge at day 7. The *P. acnes*-primed mice depleted of macrophages show phenotypes similar to naïve mice after LPS challenge [38]. They lack liver injury and 100% survive (Table 1). *P. acnes*-primed mice receiving PBS liposome, however, show the susceptibility to LPS similar to that in *P. acnes*-primed mice [38]. Thus, macrophages are necessarily required for the *P. acnes*-induced sensitization to LPS.

## 5. Importance of MyD88-IL-12-IFN-*γ* Axis for the Sensitization to LPS

It is well established that IFN-*γ* can potently prime macrophages to efficiently respond to LPS [42]. IFN-*γ*-primed macrophages produce much larger amounts of TNF-*α* and IL-6 than naïve cells [32]. Furthermore, Th1 cell differentiation occurs both in the liver and spleen after *P. acnes* treatment in a manner dependent on IL-12, a prototype cytokine for Th1 cell differentiation [32, 43]. Splenocytes and splenic CD4^+^ T cells from *P. acnes*-primed WT mice produce a large amount of IFN-*γ* but entirely not IL-4 in response to heat-killed *P. acnes* and immobilized anti-CD3 mAb, respectively [18, 32]. Besides, splenic CD4^+^ T cells from *P. acnes*-primed *I* *l*12*p*40^−/−^ mice do not differentiate into Th1 cells [18, 44]. Hepatic CD4^+^ T cells differentiate toward Th1 cells as well, which is totally inhibited by the treatment with neutralizing anti-IL-12 monoclonal antibody (mAb) [45]. IL-12 directly activates hepatic NK cells to produce IFN-*γ* [46, 47]. Furthermore, hepatic NK cells are numerically increased and acquire the high responsiveness to LPS during *P. acnes* priming phase [48]. From these observations together, one may assume the importance of IL-12-IFN-*γ* axis for the development of LPS sensitization via induction of Th1 cells. Expectedly, *P. acnes*-primed *I* *f* *n* *γ* ^−/−^ mice, *I* *l*12*p*40^−/−^ mice or mice with inherited unresponsiveness to IL-12 are resistant to LPS, in terms of lack of hypothermia, hypercoagulation or high mortality [32, 49] (Table 2). In addition, neither *I* *f* *n* *γ* ^−/−^ nor *I* *l*12*p*40^−/−^ mice form dense hepatic granulomas after *P. acnes* treatment [18, 50] (Table 2). Thus, IL-12-IFN-*γ* axis is critical for the LPS sensitization.

**Figure 5:**
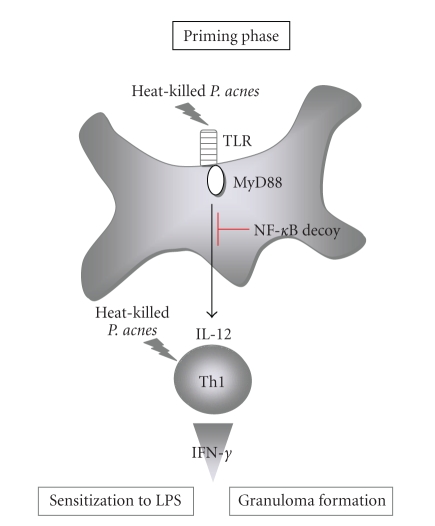
Molecular and cellular mechanisms underlying *P. acnes*-induced sensitization to LPS. After recognition of heat-killed *P. acnes*, cytoplasmic domain of TLR9 recruits MyD88 and relays a signal for nuclear translocation of NF-*κ*B, eventually resulting in various gene expressions including IL-12 production. IL-12 is involved in the development of *P. acnes*-specific Th1 cells, which produce robust IFN-*γ* in response to *P. acnes*. IL-12 also activates hepatic NK cells to release IFN-*γ*. IFN-*γ* derived from Th1 cells and NK cells sensitize mice to LPS and induce their dense hepatic granuloma formation. Administration of NF-*κ*B decoy profoundly inhibits both the LPS sensitization and the hepatic granuloma formations [16].

As they cannot actively enter into inside of cells, heat-killed *P. acnes* are likely to be recognized by extracellular sensor TLR. As expected, MyD88, which is a key signal adapter molecule of the major TLR signal pathway [2], is essentially required for the development of hepatic granulomas after *P. acnes* priming, strongly suggesting critical role of TLR/MyD88 pathway in the development of *P. acnes*-induced LPS sensitization. *M* *y* *d*88^−/−^ mice lack hepatic granuloma formation after *P. acnes* treatment, and after LPS challenge *P. acnes*-primed *M* *y* *d*88^−/−^ mice do not suffer from the mortality or liver injuries [38, 51] (Table 2, Figure 5). The MyD88-mediated pathway activates nuclear translocation of NF-*κ*B [2]. It is intriguingly to note that administration of NF-*κ*B decoy during *P. acnes* priming phase completely abrogates the hepatic granuloma formation and the sensitization to LPS in WT mice [16]. This strengthens further the importance of the MyD88-mediated pathway for the LPS sensitization. Among TLR members, TLR9 that senses bacterial unmethylated CpG DNA, but not TLR2 that recognize bacterial cell wall product peptidoglycan, was clearly verified to be required for the LPS sensitization by *P. acnes *priming [52, 53]. Indeed, *P. acnes*-primed *T* *l* *r*2^−/−^ mice are comparably susceptible to LPS as *P. acnes*-primed WT mice, although *P. acnes* possess abundant TLR2 ligands in their cell walls (52). In contrast, *P. acnes*-primed *T* *l* *r*9^−/−^ mice, like *M* *y* *d*88^−/−^mice, fail to develop hepatic granulomas and become susceptible to LPS [53]. This suggests that unmethylated CpG-DNA of *P. acnes* is pivotal for the sensitization to LPS at least by *P. acnes*-priming. Taken together, these observations strongly suggest that the MyD88-IL-12-IFN-*γ* axis plays a pivotal role in the hepatic granuloma formation and sensitization to LPS (Figure 5).

Upon challenge with TNF-*α* instead of LPS, *P. acnes*-primed WT mice show the manifestations/signs similar to those of endotoxin shock syndrome [29, 30, 32], indicating that TNF-*α* is an effector cytokine and that *P. acnes* treatment tremendously facilitates responsiveness to TNF-*α*. TNF-*α*-challenged, *P. acnes*-primed mice, but not naïve mice, suffer from hypothermia with exceptionally high plasma levels of plasma TAT and PAI-1 and show high mortality. Consistently, *P. acnes*-primed *I* *f* *n* *γ* ^−/−^ mice are resistant to TNF-*α* as well [32]. Thus, *P. acnes* treatment renders mice highly susceptible to LPS for TNF-*α* production and also to TNF-*α* itself via induction of IFN-*γ* production.

## 6. Importance of IFN-*γ* for the Systemic Endotoxin Shock Manifestations after LPS Challenge

Administration of neutralizing anti-IFN-*γ* mAb just before LPS challenge could partly rescue *P. acnes*-primed mice from hypothermia, hypercoagulation, and high mortality [32], demonstrating the importance of endogenous IFN-*γ* for the accomplishment of LPS phase as well. Taken together, IFN-*γ* is a master regulator of the systemic endotoxin shock syndrome by induction of the sensitization to LPS and activation of the LPS phase.

## 7. IL-18 Is Necessary and Sufficient for the Development of Liver Injuries

**Figure 6:**
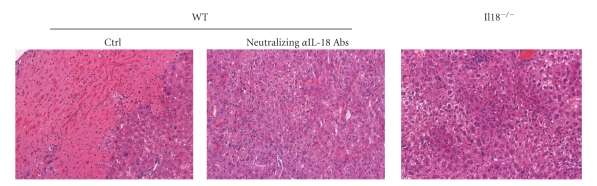
Importance of IL-18 for the development of *P. acnes*/LPS-induced liver injury.* P. acnes*-primed wild-type (WT) showed liver necrosis after LPS challenge. However, treatment with neutralizing anti-IL-18 just before LPS challenge could protect against this liver injuries [17]. Furthermore, *I* *l*18^−/−^ mice were resistant to the sequential treatment with *P. acnes* and LPS [18].

Upon LPS challenge many *P. acnes*-primed WT mice shortly died, and the surviving mice suffer from liver injuries later (Figure 6). Blockade of IL-18 or genetic depletion of *Il18* can protect against the liver damages [17, 54] (Figure 6). Upon LPS challenge *P. acnes*-primed *I* *l*18^−/−^ mice having normally dense hepatic granulomas develop the endotoxin shock syndrome comparably as *P. acnes*-primed WT mice [18]. In contrast, the surviving *I* *l*18^−/−^ mice evade the liver injuries [18] (Figure 6). These results indicate that IL-18 is necessary for the development of this liver injury. Furthermore, administration of IL-18 causes liver injuries in *P. acnes*-primed WT mice but not naïve mice [55]. Therefore, IL-18 is necessary and sufficient for *P. acnes*/LPS-induced liver injury.

IL-18 is capable of inducing hepatocytotoxic TNF-*α* directly in many cell types [13]. NK cells and Th1 cells, but not naïve CD4^+^ T cells, express IL-18R [47, 56]. During *P. acnes* priming phase, naïve CD4^+^ T cells differentiate into *P. acnes*-specific Th1 cells as described above. Therefore, IL-18 activates both NK cells and *P. acnes*-specific Th1 cells to produce robust IFN-*γ*, which in turn might fully activate Kupffer cells and hepatic macrophages to further produce TNF-*α* [54]. In addition, IL-18 has potent capacity to induce and upregulate Fas ligand expression on NK cells enough to kill Fas-expressing hepatocytes [54]. Thus, endogenous IL-18 participates in the liver injuries through induction of proinflammatory cytokines and cell death-inducing protein.

## 8. Kupffer Cells Secrete IL-18 in a Manner Dependent on TRIF and the NLRP3 Inflammasome

Many investigators use peritoneal exudate cells (PEC) prepared from the mice administered intraperitoneally with thioglycorate or bone marrow-derived macrophages (BMM) by incubation of bone marrow cells with recombinant monocyte colony-stimulating factor as conventional murine macrophages. These two types of macrophages cannot secrete IL-1*β* or IL-18 after stimulation with LPS alone. However, LPS-primed PEC or BMM can secrete robust IL-1*β* and IL-18 upon subsequent stimulation with exogenous ATP in a TLR4- and caspase-1-dependent fashion [57, 58]. From these observations the following possibilities have been believed. First, TLR4-mediated signal pathway cannot activate caspase-1. Second, ATP signaling via its cell surface receptor P2x_7_R is a central event required for the caspase-1 activation in LPS-stimulated macrophages. Third, the TLR4-mediated signal pathway is only required for induction of proIL-1*β* production. We also confirmed the absence of IL-1*β* or IL-18 release from LPS-activated PEC or BMM. In contrast to these PEC and BMM, WT Kupffer cells can release substantial amounts of IL-1*β* and IL-18 in response to LPS or synthetic lipid A (active center of LPS) alone [15, 38, 54, 59] (Table 3), strongly suggesting involvement of the TLR4 signaling in release of IL-1*β* and IL-18. As LPS-stimulated caspase-1-deficient Kupffer cells do not secrete IL-1*β* or IL-18 [11, 12], caspase-1 is an essential processing enzyme of such IL-1*β* and IL-18. In fact, western blotting analyses reveal active form of caspase-1 in the lipid A-stimulated WT Kupffer cells [38] (Table 3). Expectedly, *T* *l* *r*4^−/−^ Kupffer cells fail to activate caspase-1 upon stimulation with lipid A [38]. Thus, Kupffer cells seem to be different from PEC or BMM in the ability to activate caspase-1 upon TLR4 engagement. However, it is still to be elucidated how Kupffer cells acquire the potential to activate caspase-1 in response to TLR4 agonists alone.

**Figure 7:**
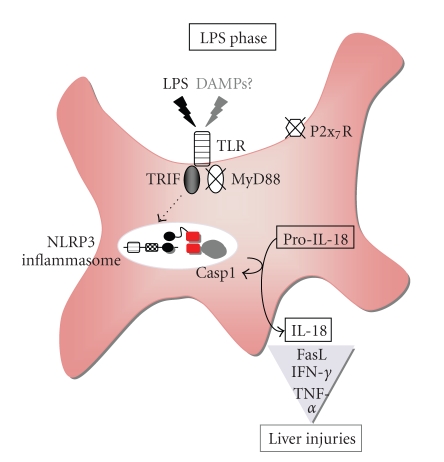
Molecular and cellular mechanisms for LPS phase. After challenge of *P. acnes*-primed wild-type mice with LPS, TLR4 is activated via TRIF for activation of NLRP3 inflammasome, which eventually leads to cleavage of procaspase-1 into its active form caspase-1. Active caspase-1, then, processes pro-IL-18 into active IL-18 for extracellular release. IL-18 upregulates both hepatotoxic Fas ligand (FasL) expression and TNF-*α* production. DAMPs and alarmin released from the injured hepatocytes might further activate the Kupffer cells, eventually resulting in the acceleration of inflammatory responses. DAMPs, damage-associated molecular patterns.

The TLR4 signaling is relayed by the MyD88- and TRIF-mediated pathways [2]. *M* *y* *d*88^−/−^ Kupffer cells stimulated with TLR4 agonists show normal caspase-1 activation [38]. As the *M* *y* *d*88^−/−^ Kupffer cells cannot produce pro-IL-1*β*, eventually resulting in lack of mature IL-1*β* secretion [38] (Table 3). In contrast to proIL-1*β*, pro-IL-18 is constitutively stored in *M* *y* *d*88^−/−^ Kupffer cells as well as WT cells [15]. Therefore, it is convincing that *M* *y* *d*88^−/−^ Kupffer cells cultured with LPS can secrete IL-18 [15, 38] (Table 3). *T* *r* *i* *f* ^−/−^ Kupffer cells show the reverse phenomena. Despite of their normal production of pro-IL-1*β* and pro-IL-18, *T* *r* *i* *f* ^−/−^ Kupffer cells cannot release IL-1*β* or IL-18 due to their inability to activate caspase-1 [38]. These results demonstrate a pivotal role of TRIF but not MyD88 in the TLR4-mediated caspase-1 activation (Table 3, Figure 7). 

*A* *s* *c* ^−/−^ Kupffer cells have the phenotype similar to *C* *a* *s* *p* *a* *s* *e*1^−/−^ cells [38, 60], suggesting that NLRP3 or AIM2 inflammasome or unidentified one that needs ASC protein (Figure 1) is involved in the caspase-1 activation. Lipid A-stimulated *N* *l* *r* *p*3^−/−^ Kupffer cells fail to secrete IL-18 or IL-1*β* [38]. Therefore, the NLRP3 inflammasome activation is necessary for the TLR4-mediated casapse-1 activation (Table 3, Figure 7).

These results cannot exclude the possibility that the TRIF-mediated pathway might cause extracellular release of ATP and that this self-derived ATP might activate the NLRP3 inflammasome in LPS-stimulated Kupffer cells [57, 58]. Unexpectedly, *P*2*x*7*r* ^−/−^ Kupffer cells show normal caspase-1 activation and normal release of IL-1*β* and IL-18 [38]. These results demonstrate the dispensability of endogenous ATP/P2x_7_R-mediated pathway for the TLR4/TRIF/NLRP3-mediated caspase-1 activation (Table 3, Figure 7).

Although we now know the importance of the NLRP3 inflammasome, the precise mechanisms by which the TRIF-mediated signal pathway activates the NLRP3 inflammasome is unclear. It is also unknown whether NLRP3 protein directly recognizes TLR4 agonists. If so, how do the TLR4 agonists translocate into the inside of Kupffer cells? Alternatively, does TRIF-mediated pathway trigger synthesis of cytoplasmic NLRP3 agonist? If so, what is the NLRP3 agonist? And, how about the molecular mechanisms for the TRIF-induced NLRP3 agonist? We need further extensive studies to address these key queries.

## 9. Requirement of NLRP3 Inflammasome Activation for the Liver Injury

The capacity to activate caspase-1 reflects on the development of liver injury [38, 60]. Expectedly, *P. acnes*-primed *T* *l* *r*4^−/−^ mice, although manifesting normal levels of hepatic granuloma formation, can avoid the liver injury after LPS challenge accompanied by lack of serum elevation of IL-18 (Table 3, Figure 7). Since they fail to develop hepatic granulomas, *P. acnes*-primed *M* *y* *d*88^−/−^ mice lack the production of robust IL-18 after *P. acnes* priming, presumably resulting in escape from the liver injury (Table 3, Figure 7). This demonstrates again requirement of MyD88 for the *P. acnes*-induced LPS sensitization. Conversely, *P. acnes*-primed *T* *r* *i* *f* ^−/−^ mice, Caspase1^−/−^ mice, *A* *s* *c* ^−/−^ mice and *N* *l* *r* *p*3^−/−^ mice all have normal dense granulomas in their livers, but fail to develop liver injury after LPS challenge, concomitant with the absence of the serum IL-18 increase (Table 3, Figure 7). *P*2*x*7*r* ^−/−^ mice have comparable phenotype as WT mice (Table 3, Figure 7), demonstrating dispensability of endogenous ATP/P2x_7_R pathway for the liver injuries. Thus, the TLR4/TRIF-mediated activation of NLRP3 inflammasome is critical for the development of the *P. acnes*/LPS-induced liver injuries via activation of caspase-1 for maturation and release of IL-18. 

## 10. Methods

### 10.1. Mice

C57BL/6 mice were purchased from Clea Japan (Osaka, Japan). Female mice (8–12-week-old) were used for this study. Mice were maintained under specific pathogen-free conditions, and received humane care as outlined in the Guide for the Care and Use of Experimental Animals in Hyogo College of Medicine.

### 10.2. Reagents

Monoclonal antibody (mA) against F4/80 of mouse macrophage was purchased from BMA (Augst, Switzerland). DAPI was from KPL (Gaitherburg, MD).

### 10.3. Treatment with P. acnes

Heat-killed *P. acnes* were labeled with or without Cy3 (GE, Buchinghamshire, UK) according to the manufacture's instruction and were injected into mice through a tail vein. At the indicated time points various tissues and tissue specimens were sampled for weighing and analysis of the cellularity by confocal microscopy, respectively.

### 10.4. Confocal Microscopic Analysis

Frozen sections of various tissues were incubated with mAb against F4/80, biotinylated antirat IgG, and then Alexa Fluor 488-conjugated streptavidin (Molecular Probes). Nuclei were stained by DAPI. The immunostaining of each section was evaluated using a laser scanning confocal microscopy [61, 62].

### 10.5. Flowcytometry [61]

Spleen cells and Kupffer cells were isolated from variously treated WT B6 mice [55]. Cells were incubated with APC-conjugated anti-F4/80 mAb.

## 11. Closing Remarks

PAMPs evoke innate immune responses by activating pattern recognition receptors (PRRs), such as TLR, NLR, and RLR. Similarly, injured host cells release endogenous “damage”-associated molecular patterns (DAMPs) that induce similar responses via recognition by PRRs [63–65]. For example, high-mobility group box1 protein (HMGB1) that is localized in the nuclei of various cell types in the steady state becomes to be extracellularly released upon stimulation of the cells with death stress. HMGB1, then, initiates innate immune responses via activating TLR4 [66]. Mitochondria are endosymbionts derived from certain bacteria during the evolution of life. Therefore, it is plausible that mitochondria possess DAMPs homologous to its ancestral PAMPs. Very recently, this was verified [67]. Mitochondrion possesses unmethylated CpG-DNA and formyl peptides similar to bacterial N-formylated proteins, which are recognized by PRRs expressed on neutrophils, TLR9, and formyl peptide receptor, respectively. Intravenous injection of the mitochondrial DAMPs causes systemic inflammatory responses and lung injuries [67]. As trauma patients have elevated serum levels of these mitochondrial DAMPs, sterile injury-induced systemic inflammatory response syndrome (SIRS), often occurring after severe trauma, might undergo in response to endogenous mitochondrial DAMPs derived from the injured cells [67]. In addition to DAMPs, self-derived “alarmin” is proposed as another potent inflaming molecules. “Alarmin” is compartmentalized in certain organelle in the steady state. Once damaged, cells begin to actively secrete “alarmin”, which in turn triggers inflammatory responses. Intraperitoneal injection of dying cells was reported to be able to trigger peritonitis with dense neutrophil recruitment in an IL-1*α*/IL-1R-dependent manner [68]. Furthermore, administration of acetoaminophen, a common antipyretic, causes massive liver injury with sterile neutrophilic inflammation in a manner dependent on IL-1*α* presumably derived from the damaged hepatocytes as well [68]. Thus, dying cell-derived IL-1*α* is regarded as alarmin. Like IL-1*α*, IL-33 is localized in the cell nuclei in the steady state and is believed to be secreted after stimulation of the cells with death stress [69]. Histone proteins derived from cell nuclei play a role as alarmin as well [70]. These endogenous DAMPs and alarmin might accelerate liver injuries induced by exogenous PAMPs and might become novel therapeutic targets for severe sepsis with organ failures.

## Figures and Tables

**Table 1:** Clinical manifestations upon LPS challenge. Naïve mice, *P. acnes*-primed mice, and *P. acnes*-primed mice depleted of macrophages (Mø) were challenged with LPS, and hypothermia, hypercoagulation, lethality and liver injuries were monitored by measurement of rectal temperature^a^, measurement of plasma TAT/PAI-1 levels^b^ and histological analyses^d^.

		Systemic alterations			Liver injury^d^
Mice		Hypothermia^a^	Hypercoagulation^b^	Lethality (%)^c^	
Naïve		−	±	0	−
*P. acnes*-primed	M*ø*-sufficient^e^	+++	+++	100	+++
	M*ø*-ablated^f^	ND	ND	0	−

^a^ +++ indicates more than 5°C reduction of rectal temperature after LPS challenge; − indicates less than 1°C of it.

^b^ +++ indicates more than 10 *μ*g/ml of plasma TAT levels; − indicates normal range of them (< 50 ng/ml); ± indicates less than 200 ng/ml.

^c^Mice were monitored for 48 h after LPS challenge.

^d^+++ indicates more than 300 IU of serum ALT levels; − indicates normal range of them (< 50 IU).

^e^ *P*. *a* *c* *n* *e* *s*-primed mice were treated twice with PBS liposome.

^f^ *P*. *a* *c* *n* *e* *s*-primed mice were treated twice with clodronate liposome to deplete macrophages.

ND; not done

**Table 2:** Importance of IL-12-IFN-*γ* axis for in vivo LPS sensitization by *P. acnes* treatment. Mice with various genotypes were sequentially administered with *P. acnes* and LPS. At day 7 after *P. acnes* priming, hepatic granuloma formation was determined by histological analyses.

*P. acnes*-primed mice	Sensitization phase	LPS phase				
	Granuloma formation^a^	Hypothermia^b^	Hypercoagulation^c^	Serum TNF-*α* ^d^	Lethality^e^	Liver injury^f^
WT	+++	+++	+++	+++	100	+++
*I* *l*12*p*40^−/−^	−	−	−	−	0	−
*I* *f* *n* *γ* ^−/−^	−	−	−	−	0	−

^a^ +++ indicates that 20% and more area of the liver section is occupied by granulomas; − indicates no granulomas.

^b^ +++ indicates more than 5°C reduction of rectal temperature after LPS challenge; − indicates less than 1°C of it.

^c^ +++ indicates more than 10 *μ*g/mL of plasma TAT levels; − indicates normal range of them (< 50 ng/mL); ± indicates < 50 ng/mL and > 200 ng/mL.

^d^ +++ indicates more than 5 ng/mL; − indicates less than 0.1 ng/mL.

^e^ Mice were monitored for 48 h after LPS challenge.

^f^ +++ indicates more than 300 IU of serum ALT levels; − indicates normal range of them (< 50 IU).

**Table 3:** Requirement of MyD88 and TRIF for LPS sensitization and Caspase-1 activation, respectively. Mice with various genotypes were sequentially treated with *P. acnes* and LPS, and liver specimens and sera were sampled for histological analyses and measurement of IL-18/IL-1*β* levels by ELISA, respectively. Kupffer cells were incubated with LPS for 4 h, and each supernatant was collected for western blotting analyses and ELISA. Naïve mice have no hepatic granulomas or injuries, and their serum IL-18 and iL-1*β* were undetectable. Naïve Kupffer cells released no IL-18 and IL-1*β*.

Genotype	Sensitization phase	Response to LPS				
		*In vivo*		LPS-stimulated Kupffer cells		
				Western blotting analyses		ELISA
	Granuloma formation^a^	Liver injury^b^	Serum IL-18/IL-1*β* ^c^(ELISA)	ProIL-1*β* production^d^	Active Casp1^e^	IL-1*β*/IL-18 release^f^
WT	+++	+++	+++	+++	+++	+++
*T* *l* *r*4^−/−^	+++	−	−	−	−	−
*M* *y* *d*88^−/−^	−	−	−	−	+++	++^*g*^
*T* *r* *i* *f* ^−/−^	+++	−	−	+++	−	−
*C* *a* *s* *p*1^−/−^	+++	−	−	+++	−	−
*A* *s* *c* ^−/−^	+++	−	−	+++	−	−
*N* *l* *r* *p*3^−/−^	+++	−	−	ND	ND	−
*P*2*x*7*r* ^−/−^	+++	+++	+++	+++	+++	+++

^a^ +++ indicates that 20% and more area of the liver section is occupied by granulomas; − indicates no granulomas.

^b^ +++ indicates more than 300 IU of serum ALT levels; − indicates normal range of them (< 50 IU).

^c^ +++ indicates more than 1000 and 50 pg/mL of serum IL-18 and IL-1*β* levels, respectively; − indicates normal range of them.

^d^ +++ indicates 10 times and more pro-IL-1*β* density in cell lysates; − indicates the absence of pro-IL-1*β*.

^e^ +++ indicates the presence of active Caspase-1 (Casp1) in supernatant; − indicates the absence of it.

^f^ +++ indicates more than 100 pg/mL; − indicates undetectable levels.

^g^ ++ indicates more than 50 pg/mL of IL-18, but undetectable IL-1*β*.

ND; not done
